# Urinary Extracellular Vesicle Protein Profiles Discriminate Different Clinical Subgroups of Children with Idiopathic Nephrotic Syndrome

**DOI:** 10.3390/diagnostics11030456

**Published:** 2021-03-06

**Authors:** Lucia Santorelli, William Morello, Elisa Barigazzi, Giulia Capitoli, Chiara Tamburello, Luciana Ghio, Barbara Crapella, Stefania Galimberti, Giovanni Montini, Marina Pitto, Francesca Raimondo

**Affiliations:** 1 Clinical Proteomics and Metabolomic Unit, School of Medicine and Surgery, University of Milano-Bicocca, 20900 Monza, Italy; luciasantorelli13@gmail.com (L.S.); e.barigazzi@campus.unimib.it (E.B.); marina.pitto@unimib.it (M.P.); 2 Paediatric Nephrology, Dialysis and Transplant Unit, Fondazione IRCCS Ca’ Granda-Ospedale Maggiore Policlinico, 20122 Milan, Italy; williammorello82@gmail.com (W.M.); chiara.tamburello@policlinico.mi.it (C.T.); luciana.ghio@policlinico.mi.it (L.G.); barbara.crapella@policlinico.mi.it (B.C.); giovanni.montini@unimi.it (G.M.); 3 Center of Biostatistics for Clinical Epidemiology, School of Medicine and Surgery, University of Milano-Bicocca, 20900 Monza, Italy; g.capitoli@campus.unimib.it (G.C.); stefania.galimberti@unimib.it (S.G.); 4 Healthcare Professional Department Fondazione IRCCS Ca’ Granda, Ospedale Maggiore Policlinico, 20122 Milan, Italy; 5 Giuliana and Bernardo Caprotti, Chairs of Pediatrics, Department of Clinical Sciences and Community Health, University of Milan, 20122 Milan, Italy

**Keywords:** Idiopatic Nephrotic Syndrome, children, extracellular vesicle, liquid biopsy, protein profile

## Abstract

Idiopathic nephrotic syndrome (INS) is the most frequent primary glomerular disease in children, displaying high grade proteinuria and oedema. The mainstay of therapy are steroids, and patients are usually classified according to the treatment response (sensitive vs. resistant). The mechanisms involved in INS pathogenesis and treatment responsiveness have not yet been identified. In this context, the analysis of urinary extracellular vesicles (UEv) is interesting, since they represent a molecular snapshot of the parental cells, offering a “fingerprint” for monitoring their status. Therefore, the aim of this study is to verify the feasibility of using UEv of INS patients as indicators of therapy response and its prediction. UEv were isolated from the urine of pediatric patients in remission after therapy; they showed characteristic electrophoresis profiles that matched specific patient subgroups. We then built a statistical model to interpret objectively each patient UEv protein profile: in particular, steroid-resistant patients cluster together with a very distinct pattern from other INS patients and controls. In conclusion, the evaluation of the UEv protein profile looks promising in the investigation of INS, showing a disease signature that might predict clinical evolution.

## 1. Introduction

Idiopathic nephrotic syndrome (INS) is the most common glomerular disease in childhood and it is characterised by proteinuria, hypoalbuminaemia, and oedema. The pathogenesis is poorly understood [1], but evidence suggests an implication of immunological mechanisms, such as B- and T-cell dysfunction [2] and a possible role of Epstein–Barr virus (EBV) infection [3]. A yet unidentified permeability factor is believed to be involved at least in patients with INS relapsing after renal transplantation [4]. 

The mainstay of therapy are steroids, and INS is usually classified according to the response to drug treatment, as steroid-sensitive (SSNS) or steroid-resistant (SRNS) [5]. At least 50% of SSNS patients will require long-term steroid or immunosuppressive treatment to maintain remission and will be further classified as steroid-dependent (SDNS). The prognosis is very different according to different subgroups, with SRNS resistant to second-line treatments progressing into end stage renal disease in virtually 100% of cases [1] and relapsing after kidney transplantation in over 50% of non-genetic cases [6]. Unfortunately, since there is a lack of established and reliable biomarkers of response, children may be exposed to unnecessary and toxic immunosuppressive cures. 

Urinary extracellular vesicles (UEv) are nanometer-sized vesicles (50–200 nm) that can originate from glomerular cells (endothelial and podocytes) and tubular epithelial cells [7]. UEv proteome is enriched in renal proteins and contains less than 3% of total urine proteins (>3000 species), depleting it from the most abundant proteins (i.e., albumin) and reducing the complexity of the urine proteome [8]. Their molecular composition depends upon the type, and even status, of the cell of origin [9]. As such, they provide an easily accessible window to monitor kidney condition. For all these reasons, they can be considered as a sort of liquid biopsy, an auspicious readout giving an insight into pathophysiological processes and events associated with the urinary system, and may be able to provide biomarkers as well as molecular indicators of the disease pathogenesis and progression [10]. 

In this contest, the protein profiling of the UEv represents a valid approach to characterise children with INS. Few studies have specifically targeted the role of UEv in INS, focusing mainly on miRNA detection [11], likely because nephrotic range proteinuria negatively influences UEv isolation and the following analysis [12]. 

Aware of the technical challenges related to the study of UEv in case of proteinuria, we approached this issue focusing on UEv isolated from the urine samples of patients in remission. Here, we investigated the role of UEv proteomes in stratifying INS-affected subjects according to the treatment response, comparing SSNS, SRNS and SDNS patients, in order to develop new tools for supporting the prediction of therapy response and its evolution. 

## 2. Materials and Methods

### 2.1. Patients and Biological Sample Collection

We performed a pilot study of UEv in pediatric patients with INS. Urine samples were prospectively collected from all consecutive INS children attending the Pediatric Nephrology Dialysis and Transplant unit of Milan. Demographic data, current and previous therapies and response to ongoing therapy were collected. Patients were classified according to the response to initial steroid administration and the need for further immunosuppressive treatment into SSNS, SDNS and SRNS, according to international consensus [1]. Steroid resistance was defined according to the Italian guidelines as the persistence of nephrotic range proteinuria after 6 weeks of therapy with prednisone (60 mg/sqm) [13]. In order to prevent interferences related to the presence of serum protein in urine, patients with significant proteinuria were excluded (urinary protein/creatinine ratio, uPr/uCr > 2 mg/mg) [14]. For this reason, SRNS patients were only included after a partial or complete response to second-line treatments and genetic SRNS with a typical persistent proteinuria were excluded. A total of 46 samples (8 SSNS, 32 SDNS, 6 SRNS), from 39 patients were collected and analysed. Table 1 summarises the main clinical characteristics of the whole cohort of INS patients. Urine samples of 4 patients were collected at different times as a follow-up (#9, 20, 23 and 17). Additionally, we included for comparison UEv samples from 7 healthy controls (HC) and 5 patients affected by Gitelman syndrome (GS), a typical tubulopathy (for further clinical information about these samples, Table S1).

Second morning urine were collected, according to the guidelines provided by the Human Kidney and Urine Proteome Project (HKUPP, available online: http:www.hkupp.org, accessed on 10 May 2018). Samples (mean volume = 20 mL) were centrifuged for sediment removal (10 min at 1000× *g*, 4 °C) within 4 h from the collection. The supernatant was supplemented with protease inhibitors (Complete, Roche) and stored at −80 °C until UEv isolation.

### 2.2. Materials

Milli-Q water was used for all solutions. Phosphate-buffered saline (PBS) was from EuroClone. Bovine serum Albumin (BSA), methanol, CAPS, ZnSO_4_ and the anti-protease inhibitor cocktail (Complete, Roche, Basel, Switzerland) were from SIGMA Chemical Co. (St. Louis, MO, USA); Hybond-ECL nitrocellulose membrane was from GE (Chalfont St Giles, Buckinghamshire, UK). NuPAGE^®^ SDS-PAGE Gel Electrophoresis System components (mini gels, running and loading buffers, molecular weight markers), SYPRO™ Ruby Protein Gel Stain and BCA protein assay were supplied by Invitrogen (Thermo Fisher Scientific, Waltham, MA, USA). Anti-tumor susceptibility gene 101 (TSG101) mouse monoclonal antibody (mAb) was purchased from Abcam (Cambridge, UK); anti-CD9 mouse monoclonal antibody was from Invitrogen (Thermo Fisher Scientific). Species-specific secondary peroxidase conjugated antibodies and ECL reagents were from Pierce (Thermo Fisher Scientific). 

### 2.3. Urinary Extracellular Vesicles (UEv) Isolation 

UEv were prepared by ultracentrifugation [15] according to HKUPP (available at: http:www.hkupp.org, accessed on 10 May 2018), with minor modifications, in order to optimise the preparation on small urine volumes. Before UEv isolation, urine samples were thawed, thoroughly vortexed while thawing and adjusted to pH 7.4, if needed. All steps were performed at 20 °C. Briefly, ZnSO_4_ 1 mM was added to the urine, samples were incubated at room temperature for 1 h and then centrifuged for 30 min at 3000× *g* at 20 °C, to reduce the Tamm Horsfall protein (THP) content [16] (data not shown). Supernatants were then further centrifuged for 15 min at 17,000× *g*, and ultracentrifuged for 70 min at 200,000× *g*; crude UEv pellets were washed in PBS under same conditions and suspended in sterile PBS with protease inhibitors. Finally, the UEv samples were stored at −80 °C until use. 

### 2.4. Nanoparticle Tracking Analysis

UEv size and concentration were measured by nanoparticle tracking analysis (NTA) using a NanoSight NS300 (Malvern Instrument Inc., Malvern, UK) equipped with a 488 nm laser and a syringe pump system, with a pump speed of 30. Before injection, UEv were diluted in sterile PBS (1:100–250). The camera operated at 30 frames per second (fps), the camera level was 13. After three technical replicates (1 min each), the resulting tracking graphs were analysed by NTA 3.2 software (dev build 3.2.16, Malvern Panalytical, Malvern, UK) with a threshold of 4. 

### 2.5. Electrophoresis and Western Blotting

Protein separation was performed with the NuPAGE^®^ electrophoresis system, using 4–12% NuPAGE^®^ and MOPS (3-(N-morpholino)propanesulfonic acid) sodium dodecyl sulfate (SDS) buffer, as described [17]. Proteins were stained by SYPRO™ Ruby Protein Gel Stain to evaluate and compare the protein profiles, or were transferred to nitrocellulose membranes using a “tank” electrophoretic transfer apparatus (Hoefer, Holliston, MA, USA), to detect typical exosome markers (TSG-101, CD9). The blots were developed as described [17]. 

### 2.6. Statistical Analysis

Protein profiles from the densitometric analysis of gels were pre-processed and analysed with the open-source R software v.3.6.0 (R Foundation for Statistical Computing, Vienna, Austria). All pixels of the gel lane in each profile were considered with the exception of those included in the THP band. Pre-processing included profile alignment, elimination of THP band pixels and normalisation (i.e., dividing gel profile pixel intensities by the sum of all the intensities of the gel profile itself). We performed an unsupervised learning hierarchical clustering analysis (HCA) to qualitatively assess the gel profiles similarity. The HCA was carried out using the complete linkage method to identify similar clusters on principal components. These components were extracted from the principal component analysis (PCA) as those that explained the maximum variance of the original independent variables. To quantify the degree of similarity between the individual gel profiles and each of the five mean group profiles taken as reference, the cosine correlation index was calculated. This measure was used to obtain the cosine angle between the directions in space of two sequences of intensity pixel. The cosine index varies from 0 (i.e., the protein profiles are completely different) to 100 (i.e., the protein profiles are identical). The highest of the five indices obtained from each patient was used to classify the whole sample of subjects in the study.

## 3. Results

### 3.1. UEv Characterisation: Nanoparticle Tracking Analysis (NTA) and Marker Enrichment

**Figure 1:**
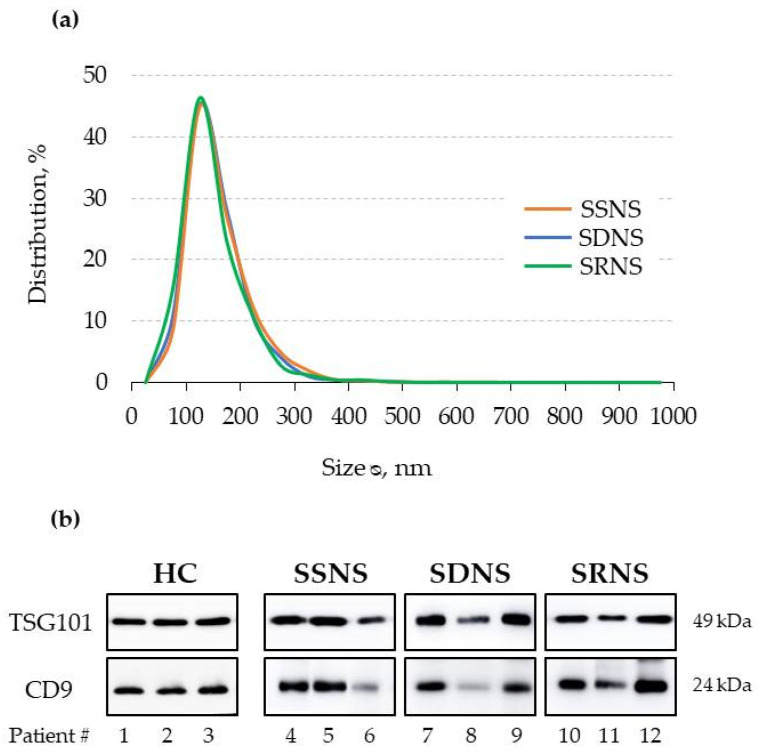
(**a**) Size distributions (%) of urinary extracellular vesicles (UEv) isolated from the urine of INS patients (NTA). Mean of 3 samples for each group; relative error for SSNS (0.74–1.13), SDNS (0.26–1.01), SRNS (0.41–0.78). (**b**) Western blot analysis of exosomal typical markers, anti-tumor susceptibility gene 101 (TSG-101) and CD9. Three representative cases for each subgroup are shown; equal protein amounts were loaded for each sample. HC, healthy controls; SSNS, steroid-sensitive; SDNS, steroid-dependent; SRNS, steroid-resistant.

UEv characterisation by NTA showed that UEv size displays a typical distribution and not significantly different among all INS groups (Figure 1a): the peak of the most represented vesicle population is 101–200 nm in diameter, as expected [18]. The detection of larger vesicles (201–300 nm diameter) is expected, considering that the urine are a complex biological matrix. UEv purity was checked by evaluating two commonly used exosomal markers, TSG101 and CD9 [19]. Immunoblotting analysis revealed that UEv obtained from all the three patient groups contain similar amounts of UEv-associated proteins (Figure 1b). Although TSG101 and CD9 are typically markers of exosomes, which by definition are less than 100 nm in diameter, we prefer the term UEv because the vesicles in our preparations had larger size. The assessment of UEv protein markers was extended to UEv isolated from all cases and demonstrated that their purity was comparable in all the preparations, although with some inter-individual variability (data not shown).

### 3.2. UEv Protein Profiling

**Figure 2:**
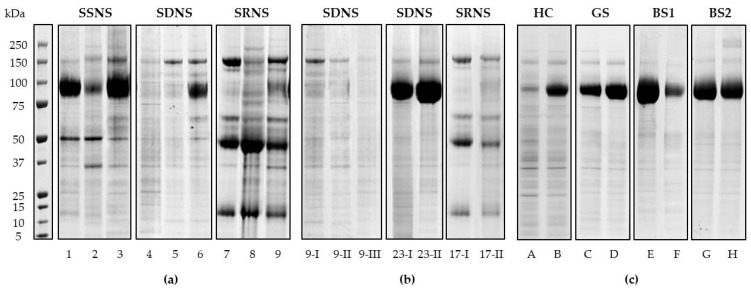
UEv protein profiles. NuPAGE^®^ 4–12% electrophoresis and Sypro Ruby protein gel staining. (**a**) Patients affected by INS: steroid-sensitive (SSSN), steroid-dependent (SDSN), steroid-resistant (SRSN); three representative cases for each patient group are shown. (**b**) UEv protein profile reproducibility: UEv isolated from the same patients at different time of collection (I, first collection; II, secondo collection; III third collection). (**c**) UEv protein profile of healthy controls (HC) and patients affected by hereditary tubulopathies (GS, Gitelman syndrome; BS1, Bartter syndrome type 1; BS2, Bartter syndrome type 2): two representative cases for each patient group are shown [17]. UEv protein profiles correspond to 3 mL of starting urine.

The protein pattern of the nanovesicles was analysed by mono-dimensional gel electrophoresis separation (1DE), followed by SYPRO™ Ruby Protein Gel Stain (Figure 2). UEv isolated from the three patient subgroups showed peculiar protein profiles: the pattern of bands is rather specific of each group, apart from THP band (visible at 80–100 kDa), which has a high interindividual variability, expression of genetic heterogeneity, as already reported [20] (Figure 2a). In particular, the SRNS group showed the most characteristic profile. Moreover, we checked the reproducibility of the protein profiles of UEv, preparing them from different urine specimens of the same patients (#9, 17, 20 and 23, see Table 1), collected some months after the first time they were collected; these UEv replicates were very similar (Figure 2b).

Furthermore, the comparison with the UEv 1DE profiles obtained from healthy pediatric controls (HC) and pediatric patients affected by hereditary tubulopathies (Gitelman and Bartter syndromes) highlighted the specificity of INS UEv protein patterns (Figure 2c). In fact, the result showed that each INS 1DE profile preserves its peculiarity and substantially differs from those of non-INS patients and healthy subjects.

### 3.3. Hierarchical Clustering and Classification 

#### 3.3.1. Qualitative Evaluation

**Figure 3:**
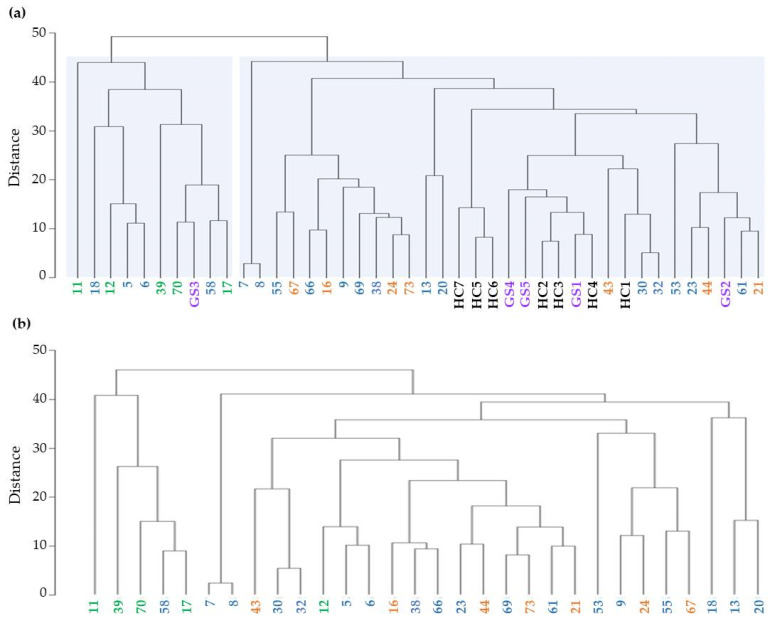
Clustering analysis of UEv protein profiles. (**a**) Clustering analysis of INS patients, healthy control (HC) and Gitelman syndrome patients (GS): SSNS (*n* = 7, orange), SDNS (*n* = 18, blue), SRNS (*n* = 5, green), GS (*n* = 5, purple) and HC (*n* = 7, black). (**b**) Clustering analysis performed within the INS patients: SSNS (*n* = 7, orange), SDNS (*n* = 18, blue) and SRNS (*n* = 5, green).

1DE protein analysis shows that UEv protein patterns match INS patient clinical classification. We verified this hypothesis assessing qualitatively gel profile similarities by a machine learning approach based on HCA. The dendrogram in Figure 3a shows the clusters of the 1DE profiles obtained from the HCA, considering the patients with confirmed INS diagnosis, Gitelman syndrome (GS) patients and healthy subjects. Some macro-separations were clearly detectable and the most evident was the one involving SRNS patients (green): they were all included in the same cluster, regardless of the type and the cumulative exposure to the ongoing therapy. SSNS (orange) and SDNS (blue) are mixed together in agreement with their clinical similarities. Patients 7 and 8 are very marginal with respect to the other SDNS patients, likely due to their clinical history of strong dependence on therapy (Table 1). Finally, the group of HC (black) and GS patients (purple) were clustered together, suggesting that GS patients do not have a specific 1DE profile. When the HCA was repeated on the restricted set of INS patients solely, we mainly obtained the same behavior with a more marked separation of the SRNS group from the other two (Figure 3b). Therefore, this approach underlines the peculiarity of the UEv protein pattern of SRNS patients and points out a level of heterogeneity between SSNS and SDNS protein profiles higher than expected, since they are clinically hardly distinguishable.

#### 3.3.2. Quantitative Evaluation

The individual values of the cosine index used to quantify the degree of similarity between the individual UEv profiles of our sample and the five average group profiles (SSNS, SDNS, SRNS, GS and HC), built as reference, are reported in Table S2 and summarized by groups in Table 2. The highest mean index in each of the five groups was associated with the subgroup the subjects belong to (Table 2, on the diagonal, in bold). In particular, the comparison with the SRNS almost consistently induced the lowest mean value, indicating that this group had a distinctive profile that differs from that of the other groups. Regarding the 8 SSNS patients’ UEv protein profiles, results show that they are in line with their reference group profile (mean = 79.1) as well. Moreover, they have a good similarity to SDNS (mean = 70.4), higher than to SRNS, GS or HC (each mean around 60). This reflects the similar clinical behavior of SSNS and SDNS patients. Overall, 43 out of the 58 subjects (79.6%) were correctly classified. No subject in the HC and GS groups was classified as INS, while only 4 SDNS of the INS patients were erroneously interpreted as HC (*n* = 3) and GS (*n* = 1). Since the main purpose of the current study was to discriminate the SRNS group from the other two, we focused only on the top-left side of Table 2 that describes the INS patients and we obtained a 78.3% (36/46) of correct identifications. Only one SRNS patient (#12) was misclassified as SDNS due to a similarity measure of 82.3%, albeit very close to the one that quantified the similarity with the SRNS reference mean profile (78.8%) (Table S2).

In addition, the process of re-classification on patients whose urine specimens has been collected several times reinforced our results. For instance, all the replicates of the patients 9, 23 and 17 (Figure 2b) were correctly classified as SDNS and SRNS, respectively, with a progressive increase of both the similarity with the true subgroups and the dissimilarity with the other groups. 

Finally, the UEv profile of a patient (ID #4, male, 13 years, uPr/uCr 1.08), initially believed to be affected by INS and later diagnosed with orthostatic proteinuria, was clearly distinguishable from the typical INS patient profiles and particularly in the dendrogram representation (Figure S1). Although this can be considered only an anecdotal observation, it may support our results.

## 4. Discussion

The present work highlights how urine, commonly considered merely a body waste, can be used much like a liquid biopsy, being readily accessible by non-invasive methods. In particular, UEv analysis constitutes a further level of investigation that best represents the cellular component of renal tissue. UEv are believed to offer a high diagnostic potential because they are enriched in renal proteins, and behave as functional snapshots of the kidneys and their state [21,22].

Given the potential offered by UEv, we explored their role as source of disease-related indicators in children with INS. Few papers addressed this topic, among which the most recent by Chen et al. focused on urinary exosomal microRNA (miR) content in INS children, showing the alteration of specific miR (miR-194-5p and miR-23b-3p) in response to treatment and suggesting that miR could be promising biomarkers for predicting severe complications [11]. At the protein level, the UEv Wilms tumor 1 (WT1) transcriptor factor, a well-known marker for differentiated podocytes, was proposed as a non-invasive biomarker for the detection of podocyte injury, predicting either therapy responsiveness or monitoring progression in patients with NS (focal segmental glomerular sclerosis and SSNS) [23,24]. Nevertheless, these studies did not consider the effect of proteinuria on UEv purification during the active state of the diseases, although they did consider the issues related to the normalization of the data. Rood et al. proposed a method based on ultracentrifugation and size exchange chromatography to overcome the problem, allowing the detection of lower abundant UEv proteins [25], but the protocol was laborious, and did not have a follow-on, at least with regard to INS. 

Therefore, since proteinuria is a negative interferer for UEv purification, we decided to investigate only patients in remission without significant proteinuria. Patients were classified according to steroid sensitivity and to the need for further immunosuppressive treatment into SSNS, SDNS, and SRNS. Specifically, we investigated if there was any correlation between the UEv protein profile and the response to initial treatment with corticosteroids, which is the main indicator of long-term prognosis, steroid-resistant patients being at increased risk of end-stage renal disease [13]. 

After having checked the quality of UEv preparations, it was evident that each patient subgroup UEv had a peculiar protein band pattern, albeit with some biological variability among samples. Furthermore, these protein profiles resulted as specific for INS, since they were different from the UEv protein patterns of non-INS patients (hereditary tubulopathies) and healthy controls [17]. In addition, we confirmed that the protein profile remained unchanged over time, indicating a good reproducibility, as shown for the UEv isolated from replicated urine collections belonging to the same patients. It could be argued that the stability of the profile over time in the same individuals may well depend on an individual reproducibility more than on a specific drug response; however, it surely helps in the definition of specific UEv profiles for each condition. These observations are consistent with both the qualitative and the quantitative statistical analysis, in which SRNS patients not only clustered together within the entire cohort but also were classified as SRNS according to high similarity score. Therefore, our model discriminates the SRNS profile well. Regarding steroid-sensitive forms, they are known to have a heterogeneous clinical course of the disease ranging from non-relapsing to severely steroid-dependent forms, which cannot be predicted a priori. The UEv evaluation does not strictly classify all the SSNS and SDNS patients, whose clinical course is very similar, but it is able to associate them with the class closest to their follow-up. Therefore, it may suggest clues to determine the propensity of some patients to evolve towards dependence.

We were able to define a standard UEv protein profile specific for the INS subgroups, which might help in the clinical monitoring of these patients. Thus, we speculate that our newborn model based on 1DE protein pattern analysis could represent a tool, able to offer a complementary confirmation of the prognosis and stratification of INS ambiguous cases. 

Obviously, we are aware that this is a pilot study and the road ahead is still long. The approach is not free from limitations: first, we need a higher number of INS samples to build a robust mean protein profile for each class. Another concern is the lack of a validation group: in fact, the enrollment of additional patients in a validation cohort would allow us to confirm both our findings and their consistency over time in an independent group. However, the fact that our findings are in agreement with the stratification by steroid response is further evidence of the reliability of the model and represent a good starting point for future in-depth analysis [26]. Moreover, the identification of specific and distinctive features within UEv protein patterns of INS patients, even if in a restricted cohort, could potentially counteract the weight of the biological variability. This should be taken into consideration when devising a possible application of the model in the clinical field. Another limitation consists in the imbalance of the distribution of patients in the three subgroups, with the relatively low number of SRNS patients. However, this reflects the typical frequency of CS response in INS children. Finally, ultracentrifugation is not available in all hospital facilities, restricting the application of this approach in clinical practice. Nevertheless, the UEv purification is necessary in this discovery phase, but it could possibly be overcome once the candidate targets are identified.

In conclusion, our strategy met the crucial clinical need to distinguish between SRNS patients from the other two subgroups. This approach might not only be used as a support tool to classify patients, predicting long-term prognosis. It may also intercept possible transitions from one INS class to another, just by looking for changes in the similarity index values over time. 

In the future, it would be interesting to assess if SRNS patients can be identified earlier, by isolating UEv from the more challenging proteinuric INS urine specimens collected at the onset of the disease, allowing for a prospective rather than a retrospective approach. Moreover, UEv may have the potential to explore INS biology and to clarify the mechanisms of steroid responsiveness [26]. To achieve this goal, further studies could focus on proteomics to uncover the emerging differences in the UEv protein profile of INS patients, pointing out any candidate markers that could be detected by a more feasible test on total urine.

## Figures and Tables

**Table 1:** Clinical characteristics of the enrolled idiopathic nephrotic syndrome (INS) patients.

Patient ID	Sex	Age * (Years)	uPr/uCr (mg/mg)	Ongoing Therapy (Months) ^§^			
				PRED	CYCL	MMF	TACR
SSNS							
16	M	5	0.13	2			
21	M	3	1.67				
24	M	4	0.27	1			
43	M	17	0.08				
44	F	16	0.13				
67	F	11	0.16				
73	M	10	0.12	1			
74	M	11	0.12	<1			
SDNS							
5	M	5	0.24	<1		33	3
6	M	10	0.14			15	
7	M	12	0.60	2		96	
8	M	5	0.23			26	6
9	M	7 (I)	0.18			26	
		+4 months (II)	0.18			30	
		+10 months (III)	0.18			36	
10	M	18	0.08			5	
13	F	4	0.19			29	
14	F	8	0.18			8	
18	M	8	0.15			28	
20	M	5 (I)	0.17			38	
		+10 months (II)	0.13			48	
23	F	2 (I)	0.34			5	
		+5 months (II)	0.30			10	
30	F	6	0.16			1	
32	F	8	0.15	2		1	
38	M	15	0.27	<1		<1	
53	M	10	0.84			49	
55	M	5	0.74		38	39	
58	M	12	0.15		32		
61	M	7	0.25			14	
66	F	6	0.14			30	
69	F	15	0.15			130	
SRNS							
11	F	14	0.10		33	32	
12	F	8	0.11		33		
17	M	14 (I)	0.44	17			6
		+8 months (II)	0.32				14
39	F	12	0.14		<1		
70	F	11	0.13				

*, age at the time of urine collection; ^§^, months of therapy at the time of urine collection; SDNS, corticosteroid-dependent; SSNS, corticosteroid-sensitive; SRNS, corticosteroid-resistant; PRED, prednisone; CYCL, cyclosporin; MMF, mycophenolate mofetil; TACR, tacrolimus; OP, orthostatic proteinuria; uPr, urinary proteins; uCr, urinary creatinine; M, male; F, female; I, first collection; II, second collection; III, third collection.

**Table 2:** Description of the similarity index by subgroup calculated vs. each subgroup reference profile °.

		Subgroup Reference Profile				
		SSNS	SDNS	SRNS	GS	HC
True Subgroup	SSNS (*n* = 8)	**79.1 (10.6)**	70.4 (11.7)	59.8 (10.6)	58.6 (15.1)	61.5 (14.0)
		**6**	2	0	0	0
	SDNS (*n* = 32)	63.6 (14.8)	**72.5 (10.2)**	59.6 (12.3)	63.0 (10.4)	65.6 (14.0)
		5	**21**	2	1 *	3 *
	SRNS (*n* = 6)	61.6 (10.2)	66.0 (14.0)	**83.4 (4.2)**	61.5 (11.9)	58.5 (12.5)
		0	1	**5**	0	0
	GS (*n* = 5)	55.8 (9.4)	69.5 (8.5)	59.3 (12.5)	**82.2 (5.2)**	72.7 (7.0)
		0	0	0	**5**	0
	HC (*n* = 7)	62.2 (4.5)	71.3 (8.5)	59.2 (3.0)	75.6 (11.0)	**85.6 (5.1)**
		0	0	0	0	**7**

°, Mean (standard deviation) of the similarity score by subgroup calculated vs each subgroup reference profile. The numbers of subject samples classified in the different subgroups based on the cosine similarity index are also reported. * Subject samples that would have been correctly classified as SDNS if the classification model had been built only with INS patients. Bold fonts represent the highest mean index for each subgroup.

## Data Availability

No new data were created or analyzed in this study.

## Supplementary Materials

The following are available online at https://www.mdpi.com/2075-4418/11/3/456/s1, Figure S1: UEv protein profile of the patient affected by orthostatic proteinuria. Table S1 Genetic data of patients affected by Gitelman syndrome. Table S2, Results of the cosine similarity index to quantify the degree of similarity between each individual gel profiles and the five mean subgroup profiles taken as reference.
