# Supplementary material for: Urinary Extracellular Vesicle Protein Profiles Discriminate Different Clinical Subgroups of Children with Idiopathic Nephrotic Syndrome
Source: Diagnostics (Basel). 2021 Mar 6;11(3):456. doi: 10.3390/diagnostics11030456 (PMC7998527; doi:10.3390/diagnostics11030456)
Supplement: Supplementary file 1 [file diagnostics-11-00456-s001.zip › diagnostics-1110989-SI.3.6/Figure S1.pdf]

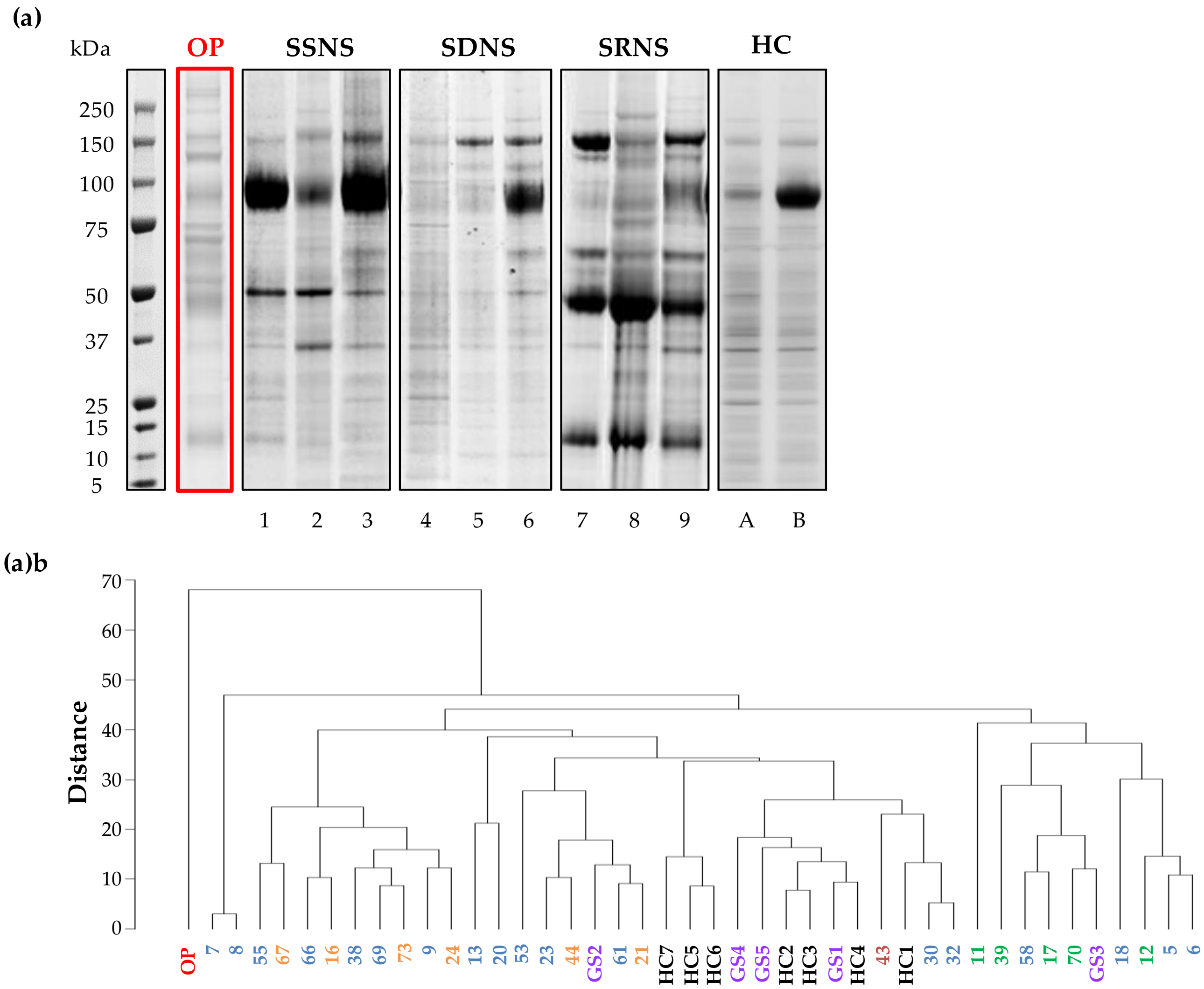

Figure S1. UEv protein profile analysis of the patient affected by orthostatic proteinuria. a) NuPAGE 4-12% electro-phoresis and Sypro Ruby protein gel staining. Patient affected by orthostatic proteinuria (OP); Patients affected by INS: steroid-sensitive (SSSN), steroid-dependent (SDSN), steroid-resistant (SRSN); healthy controls (HC). b) Clustering analysis of INS patients, healthy control (HC) and Gitelman syndrome patients (GS): SSNS (n=7, orange), SDNS (n=18, blue), SRNS (n=5, green), GS (n=5, purple) and HC (n=7, black) and OP (n=1, red).
