# Supplementary material for: Urinary Extracellular Vesicle Protein Profiles Discriminate Different Clinical Subgroups of Children with Idiopathic Nephrotic Syndrome
Source: Diagnostics (Basel). 2021 Mar 6;11(3):456. doi: 10.3390/diagnostics11030456 (PMC7998527; doi:10.3390/diagnostics11030456)
Supplement: Supplementary file 1 [file diagnostics-11-00456-s001.zip › diagnostics-1110989-SI.3.6/Table_S1.pdf]

Table S1. Genetic data of patients affected by Gitelman Syndrome [15]

Patient Code	Age	Sex	Gene Affected	Mutation at DNA Level*	Changes at protein Level	Mutation Type
5CE94	17	F	SLC12A3	c.[1844C>T]	p.[Ser615Leu]	M
				c.[1925G>A]	p.[Arg642His]	M
5ML84	27	F	SLC12A3	c.[582G>A]	p.[Gly186Asp]	M
				c.[650C>T]	p.[Arg209Trp]	M
5SA05	6	F	SLC12A3	c.[1175C>T]	p.[Thr392Ile]	M
				c.[1844C>T]	p.[Ser615Leu]	M
6CP95	16	M	SLC12A3	c.[2191G>A]	p.[Gly731Arg]	M
				c.[2661-2A>G]	p.[Ala887fs]	F
5BL94	16	M	SLC12A3	c.[2029G>A]	p.[Val677Met]	M
				c.[506-?_741+?del]	p.[Val169_Gln2474del]	LD

Notes: *: genetic variants are shown for the two alleles; del: deletion; M: missense mutation; N: nonsense mutation (stop); F: frameshift mutation; LD: large deletion; c.[?]: patient analyzed previously in a different laboratory, whose cDNA data are not available.
